# Health inequalities in childhood diseases: temporal trends in the inter-crisis period

**DOI:** 10.1186/s12939-024-02169-5

**Published:** 2024-04-17

**Authors:** Neus Carrilero, Anna García-Altés

**Affiliations:** 1 Agència de Qualitat I Avaluació Sanitàries de Catalunya (AQuAS), Barcelona, Spain; 2 Department of Medicine and Life Sciences (MELIS-UPF), Pompeu Fabra University, Barcelona, Spain; 3 Network for Research on Chronicity, Primary Care, and Health Promotion (RICAPPS) , Barcelona, Spain; 4 Research Group on Primary and Community Care in Barcelona (APICBA), Hospital del Mar Research Institute, Barcelona, Spain; 5 Network for Research On Chronicity, Primary Care, and Health Promotion (RICAPPS), Barcelona, Spain; 6 CIBER de Epidemiología y Salud Pública (CIBERESP), Madrid, Spain; 7 Institut d’Investigació Biomèdica (IIB Sant Pau), Barcelona, Spain

**Keywords:** Health inequalities, Trends, Child, Socioeconomic factors, Epidemiology, Health status

## Abstract

**Background:**

Since 2008, children in Catalonia (Spain) have suffered a period of great economic deprivation. This situation has generated broad-ranging health inequalities in a variety of diseases. It is not known how these inequalities have changed over time. The aim of the present study is to determine trends in inequalities over this period in ten relevant diseases in children according to sex and age.

**Methods:**

A retrospective cross-sectional population-based study of all children under 15 years old resident in Catalonia during the 2014–2021 period (over 1.2 million children/year) and of their diagnoses registered by the Catalan Health System. Health inequalities were estimated by calculating the relative index of inequality and time trends using logistic regression models. Interaction terms were added to test for the effects of sex on time trends.

**Results:**

Increasing significant temporal trends in inequalities were shown for both sexes in almost all the diseases or adverse events studied (asthma, injuries, poisoning, congenital anomalies, overweight and obesity), in mood disorders in boys, and in adverse birth outcomes in girls. Adjustment and anxiety and mood disorders in girls showed a decreasing temporal trend in inequalities. More than half of the diseases and adverse events studied experienced significant annual increases in inequality. Poisoning stood out with an average annual increase of 8.65% [4.30, 13.00], *p* ≤ 0.001 in boys and 8.64% [5.76, 11.52], *p* ≤ 0.001) in girls, followed by obesity with increases of 5.52% [4.15, 6.90], *p* =  < 0.001 in boys and 4.89% [4.26, 5.51], *p* ≤ 0.001) in girls.

**Conclusions:**

Our results suggest that inequalities persist and have increased since 2014. Policy makers should turn their attention to how interventions to reduce Health inequalities are designed, and who benefits from them.

**Supplementary Information:**

The online version contains supplementary material available at 10.1186/s12939-024-02169-5.

## Introduction

Spain is one of the European countries with the highest levels of inequality. According to the Oxfam report [1], Spain fares particularly poorly among European Union-28(EU-28) countries with regard to economic inequality, in terms of welfare, taxes, and workers' rights.

Children are among the groups that have suffered the most in the last decade. Since 2008, many children have faced a context of constant deprivation. Two major crises, the Great Recession of 2008 and the COVID-19 pandemic of 2020, hit family economies hard. In Catalonia, since 2014 more than 30% of children under 16 years of age have been at risk of poverty and social exclusion (with an AROPE 2020 rate averaging 377,900 children each year) [2], and Spain was second to bottom among EU-28 countries in this area in 2021 [3]. Furthermore, between 2014 and 2020, one in five children in Spain had suffered persistent poverty (defined as líving in a household below a 60% of the median value of annual disposable income (risk-of-poverty threshold) in the current year and in at least two of the preceding three years) [3].

Such widening inequalities raises concern about their impact on the health of the most vulnerable sectors of society, such as children [4, 5]. Socioeconomic inequalities in childhood affect both the present and the future health status of individuals, and do so systematically in all pathologies, in terms of both their development and their severity [6–9]. Despite the strength of the existing evidence, knowledge of inequalities caused by childhood diseases over time in Spain remains limited because of the few longitudinal studies [10]. Other European countries have recently carried out longitudinal studies assessing the impact of childhood adversity [11], socioeconomic position (SEP) [12, 13] or even foetal environmental factors [14] on health outcomes, and the cumulative effect of these factors on future development.

In Catalonia, no data are available on the impact of the economic context on inequalities in health in childhood during this inter-crisis period. The aim of this study is assess temporal trends in socioeconomic inequalities for relevant diseases and adverse events in childhood during the 2014–2021 period, according to sex and age, in more than 1.8 million children in Catalonia.

## Methods

### Context

Healthcare in Catalonia is provided by tax-funded National Health System. All residents (7,739,758 as of 2021) are granted universal public healthcare coverage by law. The use of publicly funded healthcare services is free; the sole exception is drug prescription, which is provided based on a co-payment system. Each resident is assigned a personal healthcare ID which can be used to trace their use of healthcare services.

### Study design and population

Population-based study using pooled data from all children under 15 years old resident in Catalonia during the 2014–2021 period, forming a sample of 1,815,465 unique individuals. The cutoff at 15 years old aligns with the conclusion of the pediatric stage and the transition to a general practitioner during adulthood.

### Data collection

We used two different public healthcare databases, sharing a common ID that facilitates the integration of different sources of information at the individual level.

The Central Registry of Insured Persons (RCA, Catalan acronym), is an administrative public registry from the Catalan Department of Health that includes all persons who benefit from public healthcare coverage. From it we obtain the reference population for each year in the 2014–2021 period. This registry collects and updates sociodemographic and socioeconomic data annually, including the income level, employment status, and Social Security benefits received by each individual, and these data are routinely used to calculate insurees’ pharmaceutical co-payment levels. All dependent individuals (i.e., descendants) are associated with a legal guardian, and they are assigned the same socioeconomic consideration.

The Registry of the Minimum Basic Dataset (CMBD, Catalan acronym) is an administrative public healthcare register containing detailed information on sociodemographic characteristics and medical diagnoses (coded using the International Classification of Diseases, 9th Edition). The CMBD individually encodes all contacts with the various levels of the public healthcare system: primary care, hospital care, emergency, mental health, and long-term care services. All the diagnostic codes registered in the CMBD during the 2014–2021 period were compiled to create the main data set, which was then linked to the RCA.

### Variables

The dependent variables were 10 diseases and adverse events from different aetiologies and organic systems of the child population of Catalonia. This selection includes the most prevalent pathologies and adverse events and the ones that showed the most inequalities in the Catalan population in a previous analysis [6]. Each disease variable was constructed selecting diagnostic codes based on clinical criteria (see Supplementary Table 1). Disease variable was treated as a dichotomous variable: having at least one medical diagnosis, being registered on the CMBD, included in one of the specified diseases during the year.

The independent variables were Socioeconomic Position (SEP), age, and sex. SEP was proxied by the pharmaceutical co-payment level of one of the child’s parent or guardian, obtained from the RCA registry. The pharmaceutical co-payment system uses information on income level, employment status and Social Security benefits (especially to detect individuals without any income from work or receiving welfare support due to a family environment at risk of social exclusion or poverty) and defines the following four SEP categories: “Very low” (person receiving welfare support or without any member of the household employed), “Low” (< €18,000/year), “Medium” (€18,000–€100,000/year), and “High” (> €100,000/year). This classification has been used in several previous studies [6, 15–17]. We excluded 4838 children (0.27% of all individuals) due to missing data in the SEP variable.

In accordance with the natural development of each disease and adverse event, different age limits are assigned; diseases appear at different ages, so children at risk should be considered accordingly. To establish the age limits for each disease, we calculate prevalences by age for all children (see Supplementary Fig. 1). For adverse birth outcomes and congenital anomalies we included children from newborns up to the age of two. For obesity, overweight and mental health disorders (adaptive and anxiety disorders and mood disorders) children from the age of five upwards were considered. For the other diseases and events all ages were included, from 0 to 14 years old.

### Statistical analysis

The study was carried out in three stages. First, we performed a descriptive analysis of the entire child population. Second, we assessed SEP inequalities by sex and year for each disease. Finally, we estimated inequalities and time trends according to sex, differences according to sex and average annual changes in inequalities.

Initially we described and tested sex differences in the sociodemographic and clinical variables of all the child population. We also calculated the prevalence for each disease and adverse event by year and sex, taking the population at risk according to the age límits established for each one.

Then, to measure the SEP inequalities in each disease and event we computed the regression-based relative index of inequality (RII). RII is a relative summary measure that takes account of the size of the population and the relative disadvantage for every stratum of SEP, rather than comparing only the two most extreme SEP levels. The major strength of the RII is its use of the socioeconomic weighted rank (Ridit-score), which allows an accurate comparison between populations [18].

Calculation of the RII involves the following steps: 1) determination of the prevalence of each disease occurence by SEP and sex; 2) calculation of the Ridit-score: ranking SEP levels by hierarchical assignment of a value from 0 (lowest SEP) to 1 (highest SEP) according to its proportional size in the population by linear regression. The population of each SEP level is assigned a Ridit-score based on the mid-point of its cumulative distribution in the given category. For instance, if the percentage of the sample in the lowest SEP category was 10%, the Ridit-score for this category would be 5% (10%/2). If the percentage of the sample of the second lowest SEP category was 30%, the Ridit-score would be 25% (10% + 30%/2). And finally, 3) inclusion of the Ridit-score (as a weighted SEP) as exposure and disease as a dichotomous dependent variable in an age-adjusted generalised linear model (log-binomial regression) with a logarithmic link function for each disease and sex, as follows (Model 1):

1 $${\text{log}}\left({\text{Y}}\right)=\alpha +{\beta }_{1}Riditscore+{\beta }_{2}year+{\beta }_{3}age+\epsilon$$

The coefficient β_*1*_ is the coefficient of interest and expresses the RII when the link function is log, Y = 1 for disease occurrence and Y = 0 for no occurrence. The highest SEP category was used as a reference. The RII is interpreted as the risk ratio of an outcome between the most and least advantaged in the social hierarchy [18].

Furthermore, to assess temporal trends of the RII we added a two-way interaction term in the model between the Ridit-score and year for each sex (Model 2).

2 $${\text{log}}\left({\text{Y}}\right)=\alpha +{\beta }_{1}Riditscore+{\beta }_{2}year+{\beta }_{3}age+{\beta }_{4}Riditscore\#year+ \epsilon$$

To determine the sex effect on SEP inequality trends, we added a three-way interaction term to the previous model.

3 $${\text{log}}\left({\text{Y}}\right)=\alpha +{\beta }_{1}Riditscore+{\beta }_{2}year+{\beta }_{3}age+{\beta }_{4}Riditscore\#year+{\beta }_{5}Riditscore\#year\#sex+\epsilon$$

A positive and significant result of the three-way interaction term would suggest a larger increase in the RII trend in girls than in boys during the study period [19]. We also calculated the temporal trend for each age category following the same method as with sex, replacing the sex variable with age in the model.

Finally, we estimated the average annual change in inequalities for each disease and sex for the 2014–2021 period. We did so by computing a variance-weighted least squares model for each disease and sex using the RII (as the dependent variable) on time (8 years). The RII was rescaled using a logarithmic function. The precision estimates of the RII (inverse of the standard error) were taken as weights. Hence, we calculated the marginal effects on the model; the predicted change in the RII caused by a unit change in year while all other variables remained constant. For a better interpretation of the results, we exponentiate the coefficient, subtract one from its number, and multiply by 100. This gives the percent increase (or decrease) in the response for every one-unit increase in the independent variable [20]. We also calculated the average annual change for age categories following the same method as with sex, replacing the sex variable with age in the model.

All the analyses were performed using the STATA IC/15.1 and software modules RIIGEN for RII.

All the estimates are accompanied by their 95% confidence intervals (CI) and the associated *p*-value.

## Results

### Sociodemographic and clinical characteristics

The dataset included 1,815,465 unique individuals (51.41% boys) for the entire study period, with a mean number of 619,886 (SD 11,791) boys and 584,457 (SD 11,072) girls per year. Sociodemographic and clinical characteristics by sex are displayed in Table 1. SEP and age did not show differences in distribution by sex. In each year of the period, almost 5% of the children presented very low SEP (4.98% (SD 0.43) in boys and 4.93% (SD 0.44) in girls). With regard to diseases and adverse events, boys suffer more from asthma, bronchitis, injuries and obesity than girls.

**Table 1:** Sociodemographic and clinical characteristics of the entire child population per year by sex, Catalonia 2014–2021

	**Boys**	**Girls**	
	**% (SD)**	**% (SD)**	***p***-value^c^
**Age (years old)**			
< 2	13.29 (1.22)	13.16 (1.20)	0.411
2–5	18.75 (0.98)	18.81 (1.02)	0.544
6–10	41.22 (1.01)	41.22 (0.96)	0.502
11–14	26.74 (1.56)	26.82 (1.60)	0.539
**SEP**			
Very low	4.98 (0.43)	4.93 (0.44)	0.410
Low	57.39 (2.69)	57.32 (2.67)	0.480
Medium	36.56 (2.58)	36.66 (2.60)	0.530
High	1.07 (0.22)	1.09 (0.20)	0.573
**Diseases/adverse events**^a^			
Asthma	5.10 (0.46)	3.43 (0.65)	≤ 0.001
Bronchitis	11.74 (1.43)	9.58 (1.61)	0.007
Injuries	18.52 (2.06)	15.27 (2.35)	0.005
Poisoning	0.33 (0.11)	0.31 (0.13)	0.422
Mood disorders	1.29 (0.21)	1.63 (0.07)	1.000
Adjustment and anxiety disorders	2.81 (0.20)	2.86 (0.58)	0.583
Congenital anomalies	10.03 (1.44)	9.00 (1.58)	0.098
Adverse birth outcomes	1.66 (0.12)	1.66 (0.09)	0.465
^ b^Overweight (from 5 to 14 years)	5.16 (1.07)	5.63 (0.99)	0.810
Obesity (from 5 to 14 years)	5.30 (0.51)	3.90 (0.67)	≤ 0.001
**Individuals (N, SD)**	619,886 (11,791)	584,457 (11,072)	≤ 0.001

^a^Diseases/adverse events included: From 0 to 14 years: asthma, bronchitis, injuries and poisoning. From 0 to 2 years old: congenital anomalies and adverse birth outcome (short gestation, low birth weight and fetal growth retardation). From 5 to 14 years old: mood disorders, adjustment and anxiety disorders, overweight and obesity

^b^Overweight does not include obesity

^c^Test for differences of means between sexes, t-test for continuous variables and χ2 for categorical variables. Significant at 95% confidence level

### Prevalence trends

**Fig. 1:**
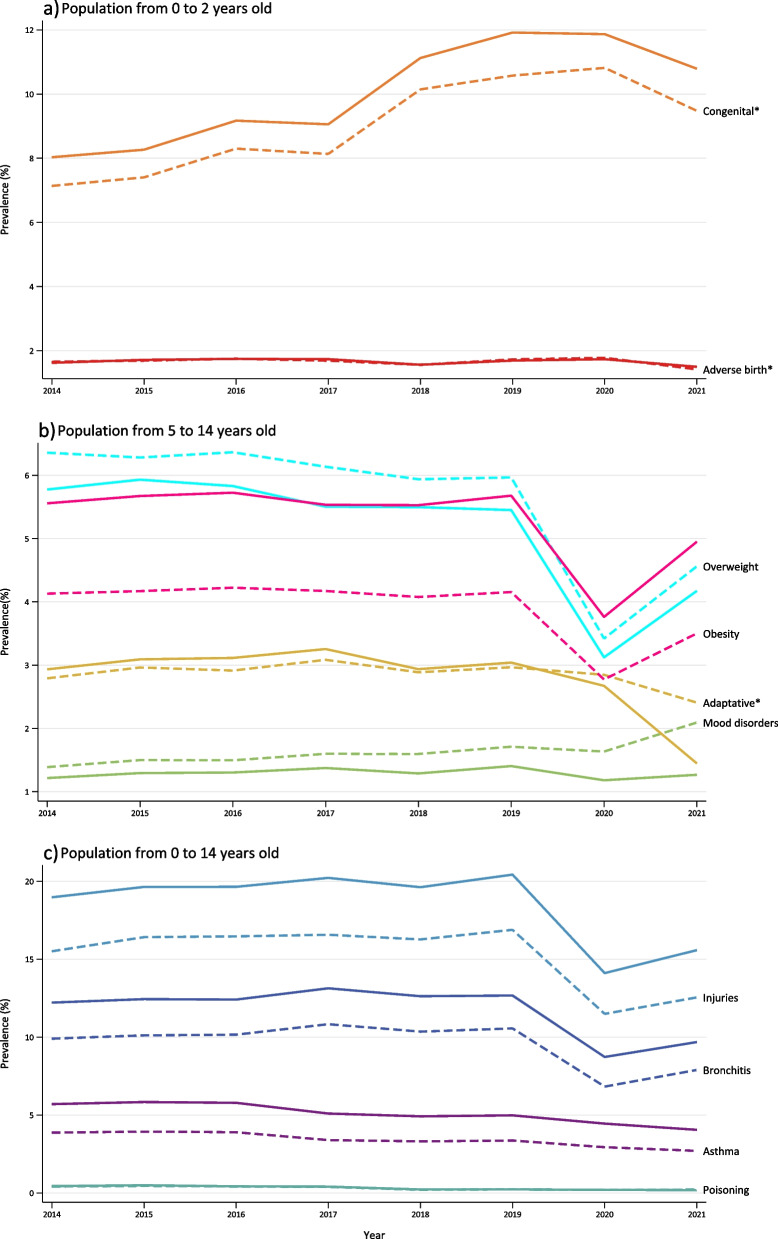
Prevalence trends for diseases and adverse events by sex according to age, 2014–2021 Catalonia. (Boys: straight lines; Girls: dashed lines).  Note: *Congenital: congenital anomalies; Adverse birth outcomes: short gestation, low birth weight, fetal growth retardation; Adaptative: adaptative and anxiety disorders.

The prevalence trends of diseases and adverse events by age group are displayed in Fig. 1. Injuries and bronchitis stand out as the most prevalent in almost every year in the study period, meaning that they had affected more than 10% of all children from 0 to 14 years old. Congenital anomalies rose steadily, reaching their maximum prevalence of 11.92% in boys and 10.82% in girls in 2020 among the 0 to 2 years age group. From 2020 onwards, disease trends were impacted by the COVID-19 pandemic and lockdown. Certain diseases and adverse events fell notably in 2020 but recovered slightly afterwards. The overall decreases in the 2019–2021 period were -22.27% in bronchitis, -29.22% in injuries, -47.02% in anxiety disorders, -13.29% in overweight and -1.97% in obesity. In contrast, mood disorders increased by 4.72% after COVID-19.

### Trends in inequalities

All diseases and adverse events studied in each year of the period and in both sexes showed significant inequalities, the sole exceptions being poisoning in 2015 in boys and congenital anomalies in 2016 in girls. Obesity was the disease with the highest inequalities, specially in girls (for specific RII values, see Supplementary Table 2).

**Fig. 2:**
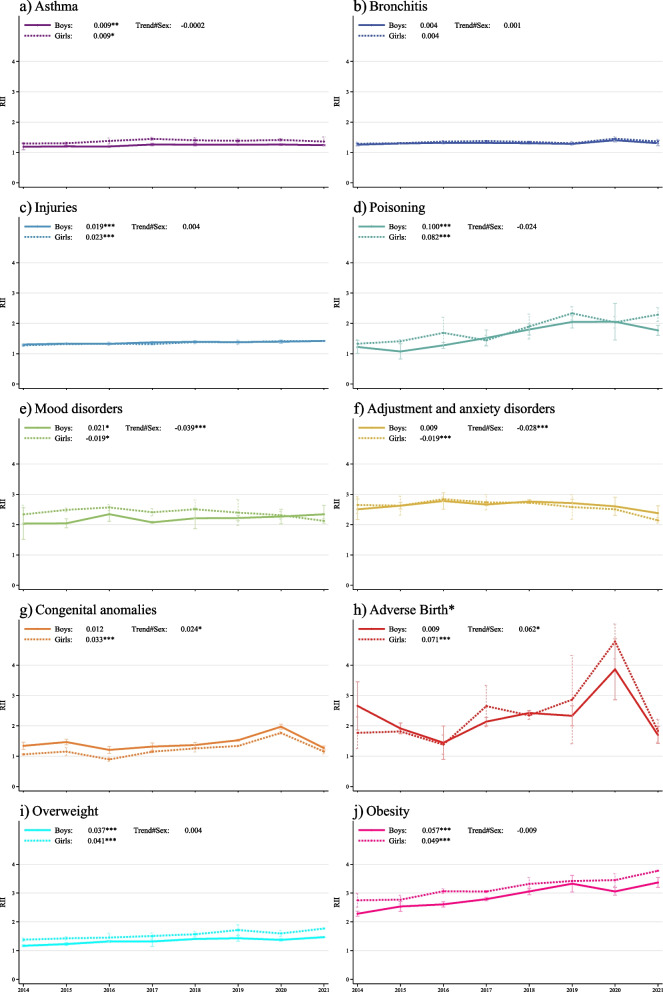
Relative index of temporal trends in inequality (two-way interaction) by sex and sex effect trend#sex (three-way interaction) by disease. (Boys: straight lines; Girls: dashed lines).  The effect of sex on trends in inequalities was significant in four diseases. Girls showed a stronger upward trend than boys in congenital anomalies (β Ridit#year#sex  = 0.024, p  = 0.003) and in adverse birth outcomes (β Ridit#year#sex  = 0.062, p  = 0.003). In contrast, girls showed a downward trend compared with boys in mood disorders (β Ridit#year#sex  = -0.039, p  = 0.010) and in adjustment and anxiety disorders (β RiditI#year#sex  = -0.028, p  = 0.010).

Note: Temporal trend: Boys and Girls; test for the temporal trend of the RII for each sex assessed adding a two-way interaction term between the Ridit-score and year. Trend#Sex; test for the sex effect in the temporal trend of the RII, assessed by adding a two-way interaction term between the Ridit-score and year and a three-way interaction term between the Ridit-score, year and sex.

Inequalities showed a significant upward temporal trend in almost all diseases and adverse events for both sexes: asthma, injuries, poisoning, congenital anomalies, overweight and obesity, and in mood disorders only in boys, while adverse birth outcomes only in girls. Inequalities presented falling temporal trends in adjustment and anxiety disorders and mood disorders in girls. Figure 2 presents the RII temporal trend for each disease and adverse event by sex and also includes coefficients and p-values of the temporal trends for each sex and for the interaction trend in inequalities between sexes.

Results for the trends in inequalities according to age revealed an inequality gradient as children grow older in asthma, bronchitis and injuries (see Supplementary Table 3 for detailed values). The effect of age on inequalities over time (three-way interaction term) exhibited a significant positive coefficient in asthma (β_Ridit#year#age_ = 0.022, *p* ≤ 0.001), bronchitis (β_Ridit#year#age_ = 0.008, *p* ≤ 0.001) and injuries (β_Ridit#year#age_ = 0.005, *p* ≤ 0.001). On the other hand, it fell in mood disorders (β_Ridit#year#age_ = -0.028, *p* = 0.013) and obesity (β_Ridit#year#age_ = -0.018, *p* = 0.010).

Diseases and adverse events considered differed according to age groups: 0 to 14 years old: asthma, bronchitis, injuries and poisoning; 0 to 2 years old: congenital anomalies and adverse birth outcomes (gestation, low birth weight and fetal growth retardation); 5 to 14 years old: mood disorders, adjustment and anxiety disorders, overweight and obesity (overweight does not include obesity).

Statistically significant levels: *** p-value ≤ 0.001, ** *p*-value ≤ 0.050, * *p*-value ≤ 0.010.

### Average annual change in inequalities

More than half of the diseases and adverse events studied presented significant annual increases in the RII during the 2014–2021 period in both sexes (see Table 2). Poisoning presented an average annual increase of 8.65% [4.30, 13.00], *p* ≤ 0.001 in boys and 8.64% [5.76, 11.52], *p* ≤ 0.001) in girls, followed by obesity, 5.52% [4.15, 6.90], *p* =  < 0.001 in boys and 4.89% [4.26, 5.51], *p* ≤ 0.001) in girls. Of note, congenital anomalies presented a far higher mean annual increase in girls than in boys: 5.92% [1.21, 10.64], *p* = 0.014 vs. 2.01% [-2.90, 6.92], *p* = 0.423.

**Table 2:** Estimated mean annual change in RII (mean margin effects) by disease and sex

	**Boys**		**Girls**	
**Disease/adverse event**^a^	**Estimate (%) (95% CI)**	***p***-value^c^	**Estimate (%) (95% CI)**	***p***-value^c^
Asthma	0.86 ( 0.29, 1.44)	0.003	1.42 ( 0.86, 1.98)	≤ 0.001
Bronchitis	0.54 ( -0.31, 1.40)	0.213	0.95 ( -0.07, 1.96)	0.068
Injuries	1.15 ( 0.84, 1.46)	≤ 0.001	1.66 ( 1.28, 2.03)	≤ 0.001
Poisoning	8.65 ( 4.30, 13.00)	≤ 0.001	8.64 ( 5.76, 11.52)	≤ 0.001
Mood disorders	1.94 ( 0.18, 3.70)	0.031	-2.18 ( -3.38, -0.98)	≤ 0.001
Adjustment and anxiety disorders	-0.32 ( -2.06, 1.43)	0.723	-3.28 ( -5.37, -1.19)	0.002
Congenital anomalies	2.01 ( -2.90, 6.92)	0.423	5.92 ( 1.21, 10.64)	0.014
Adverse birth outcomes	1.18 ( -6.83, 9.20)	0.772	7.35 ( -1.48, 16.18)	0.103
Overweight^b^	2.77 ( 2.15, 3.38)	≤ 0.001	3.58 ( 2.98, 4.18)	≤ 0.001
Obesity	5.52 ( 4.15, 6.90)	≤ 0.001	4.89 ( 4.26, 5.51)	≤ 0.001

mean percentage change in the RII for each one-unit increase in the year variable

^a^Diseases/adverse events included: From 0 to 14 years old: asthma, bronchitis, injuries and poisoning. From 0 to 2 years old: congenital anomalies and adverse birth outcomes (short gestation, low birth weight and fetal growth retardation). From 5 to 14 years old: mood disorders, adjustment and anxiety disorders, overweight and obesity

^b^Overweight does not include obesity

^c^Significant at 95% confidence level

In contrast, we found significant mean annual reductions in the RII only in girls in two pathologies: mood disorders -2.18% [ -3.38, -0.98], *p *≤ 0.001 and adjustment and anxiety disorders -3.28% [-5.37, -1.19], *p* = 0.002.

At older ages (from 5 to 14 years old), a significant increase in inequalities over the period was found in almost all the diseases and adverse events studied, particularly in asthma, injuries, poisoning, overweight and obesity (see Table 3). Overweight presented similar mean annual increases in both age categories: 3.29% [2.72, 3.86], *p* =  < 0.001 in the 6–10 age group and 3.47% [2.85, 4.09], *p* =  < 0.001 in the 11–14 age group. Inequalities in obesity rose more in the 6–10 age group (5.30% [4.20, 6.41], *p* =  < 0.001) than in the 11–14 age group (2.42% [1.30, 3.55], *p* =  < 0.001).

**Table 3:** Estimated mean annual change in RII by disease/adverse event and age

Disease/adverse event^a^	Age	Estimate (%) (95% CI)	*p*-value^c^
Asthma	< 2	-2.91 ( -3.90, -1.91)	≤ 0.001
	2–5	-1.08( -2.69, 0.53)	0.189
	6–10	1.83 ( 1.28, 2.39)	≤ 0.001
	11–14	1.92 ( 1.07, 2.76)	≤ 0.001
Bronchitis	< 2	-1.99 ( -3.77, -0.21)	0.028
	2–5	-0.42 ( -1.84, 1.00)	0.562
	6–10	0.68 ( 0.17, 1.19)	0.009
	11–14	-0.76 ( -2.58, 1.07)	0.417
Injuries	< 2	0.94 ( -0.56, 2.44)	0.221
	2–5	0.46 ( 0.07, 0.85)	0.022
	6–10	2.02 ( 1.39, 2.65)	≤ 0.001
	11–14	1.36 ( 0.83, 1.88)	≤ 0.001
Poisoning	< 2	13.96 ( 2.44, 25.49)	0.018
	2–5	9.93 ( 4.23, 15.63)	0.001
	6–10	7.18 ( 3.12, 11.24)	0.001
	11–14	8.12 ( 5.96, 10.28)	≤ 0.001
Mood disorders	6–10	3.41 ( 1.17, 5.65)	0.003
	11–14	-2.15 ( -3.34, -0.96)	≤ 0.001
Adjustment and anxiety disorders	6–10	0.44 ( -1.52, 2.40)	0.660
	11–14	-2.63 ( -4.71, -0.56)	0.013
Congenital anomalies	< 2	6.31 ( 1.66, 10.96)	0.008
Adverse birth outcomes	< 2	2.03 ( -5.68, 9.75)	0.605
Overweight^b^	6–10	3.29 ( 2.72, 3.86)	≤ 0.001
	11–14	3.47 ( 2.85, 4.09)	≤ 0.001
Obesity	6–10	5.30 ( 4.20, 6.41)	≤ 0.001
	11–14	2.42 ( 1.30, 3.55)	≤ 0.001

mean percentage change in the RII for each one-unit increase in the year variable

^a^Diseases/adverse events included: From 0 to 14 years old: asthma, bronchitis, injuries and poisoning. From 0 to 2 years old: congenital anomalies and adverse birth outcomes (short gestation, low birth weight and fetal growth retardation). From 5 to 14 years old: mood disorders, adjustment and anxiety disorders, overweight and obesity

^b^Overweight does not include obesity

^c^Significant at 95% confidence level

Poisoning presented a mean annual increase in inequalities across all age categories, from 13.96% [2.44, 25.49], *p* = 0.018 in the 0–2 years group to 8.12% [5.96, 10.28], *p* =  < 0.001 in the 11–14 years group.

Two diseases showed reductions in mean annual inequalities in the 11–14 age group: mood disorders (-2.15% [-3.34, -0.96), *p* = 0.003) and adjustment and anxiety disorders (-2.63% [-4.71, -0.56], *p* = 0.013).

## Discussion

Two main findings emerge from this study. The first is that socioeconomic inequalities are highly prevalent in all the diseases and adverse events studied, in all the years included and in both sexes. The second is that these inequalities are increasing over time in almost all the diseases and events, albeit to varying extents.

The COVID-19 pandemic affected children’s health through changes in lifestyle and the creation of barriers that hindered access to the healthcare system [5]. Our findings are consistent with to those of other studies reporting that lockdown reduced physical activity and social relationships [21], and in turn increased mental health problems [22] and obesity [23, 24]. Moreover, the restricted access to healthcare lowered rates of disease detection, particularly in childhood. This is clearly the case for overweight and obesity, as child surveillance programs were disrupted during the pandemic period. Pediatric primary care contacts in Catalonia fell by 20.6% in 2020 compared to 2019, with an even greater fall of 24.38% in early childhood (0 to 5 years old). These barriers remained in place at the end of 2021, when face-to-face visits had not yet returned to pre-COVID-19 levels [25]. Other diseases and adverse events, such as injuries and bronchitis, with an acute profile, were more likely to fall in prevalence due to the reduction in activity caused by social distancing restrictions, school closures, and lower levels of physical exercise [26].

Contrary to expectations, mental health disorders presented a variety of trends. While the prevalence of anxiety disorders fell from 2019 to 2021, the prevalence of mood disorders did not, particularly in girls. Mental health disorders reached a peak as a consequence of the pandemic measures, especially in children and adolescents. However, the effects were noted at a later stage, likely due both to difficulties of access to healthcare and to the delayed impact of post-traumatic disorders [22, 27].

Regarding childbirth, postpartum and the perinatal period, the rising prevalences of inequalities may be attributed in part to the decline in surveillance visits during pregnancy in the COVID-19 period [25], although the growth dynamics were already noticeable pre-pandemic.

Explaining the social distribution patterns of disease in childhood is complex. To establish the epidemiological behaviour of a disease, prevalences and inequalities over time must be assessed together. Having both information helps to understand the behaviour and social distribution of the disease. In general, inequalities increased in almost all the diseases and adverse events studied from 2014 to 2021. However, in spite of the impact of COVID-19, the prevalence of some diseases and adverse events has remained stable over time (e.g., bronchitis and poisoning) and even fell in some cases (e.g., asthma and overweight). Some studies echo this phenomenon in childhood, which has been termed the “inequality paradox”: namely, while the burden of disease and adverse events remains the same or decreases over time, inequalities widen [28, 29]. Our results reflect this paradox in asthma, injuries, poisoning, overweight and obesity. However, these findings may not be extrapolable to other contexts, as these prevalences are highly dependent on the degree of investment of the welfare state, public policies, and the social inequalities in each geographical area.

The prevalence of obesity among children in Spain is among the highest in Europe [30, 31]. It has fallen slightly in recent years, although the inequalities associated with it have not [32]. Temporal trends in inequalities are particularly high for overweight and obesity, increasing an average of 6% in boys and 5% in girls – one of the largest increases among the diseases studied. Absolute inequalities in girls were slightly higher than in boys over the whole period. A similar inequality paradox has been detected in other contexts, with a widening gap between socioeconomic levels despite an overall downward trend or a trend towards stability in prevalences [12, 30, 32, 33]. More attention should be paid to the socioeconomic level of the family as it is one of the most robust driving determinants of overweight, and especially of obesity [6, 12, 31, 32, 34].

Injuries and poisoning are among the pathologies and adverse events that presented a significant increase in inequalities over time in both sexes.

Injuries may have different drivers based on social inequalities, such as adult behaviour, mother’s labour status and age, injuries of previous siblings, household conditions, overcrowding and the safety of the environament, all of them unpredictable or precarious in economically deprived families [35–38]. Studies assessing trends in injuries found in other settings that while the overall incidence has fallen, differences between socio-economic levels have widened over time, especially with regard to burns [10, 39] and traffic accidents [40]. Our results show that poisoning presented a major upward trend in inequalities, with a mean annual increase of 8% in both sexes, although prevalences remained stable over time. The circumstances of poisoning vary greatly according to the age of the child. In early childhood the causes are mainly domestic or accidental [10, 41], in adolescence they are more often alcohol-related or intentional [42]. Similar contexts find similar results; poisoning was more frequent in more deprived households and the gap between socio-economic levels has widened over time [35, 41, 42]. Currently, poisoning appears to be one of the most sensitive indicators of deprivation in childhood, accounting in Catalonia and other settings for up to 30% of all adverse events due to external causes [6, 10].

The prevalence of congenital anomalies increased notably, as did their impact on inequalities over time. Our findings estimate a 6% annual increase in the RII in girls (among the largest) and a 2% increase in boys. The trend of adverse birth outcomes (mainly low birth weight) on inequalities increased over time, particularly in girls. Since the 2008 recession, the low birth weight trend has increased, especially at lower socioeconomic levels and in deprived contexts [43, 44]. Although both congenital anomalies and adverse birth outcomes have a large intra-group variability in terms of severity, these results should raise considerable concern regarding the conditions of gestation and prenatal care in families of low socioeconomic level. It is acknowledged that socioeconomic factors [14, 45] and use of drugs and alcohol during pregnancy [46, 47] present strong correlations with congenital anomalies and adverse birth outcomes. Indeed, adverse birth outcomes have been found to be more prevalent among pregnant women of low socioeconomic status in Catalonia [47, 48].

We observed a decrease in inequalities over time in mood disorders and in adjustment and anxiety disorders in girls. This pattern can be attributed to the effects of the pandemic in 2020. First, the treatment of mental health illnesses is one of the areas in which the private health sector has a strong presence [49]. Hence, in times of economic crisis, families rely more on the public health services, and so mental health issues at all SEP levels are more visible in the public health records. Second, the impact of lockdown on mental health in adolescents was more pronounced in girls, who experienced higher levels of emotional dysregulation, lower self-esteem and more depression [50] [51]. Interestingly, this could explain both the increase in the prevalence of mood disorders and the reduction in inequalities. Equally, adjustment and anxiety disorders, which have a more acute profile, seem to have had a similar impact at all socioeconomic levels, with reductions in both prevalence and impact on inequalities. Further studies are now needed to improve our understanding of this pattern and of the differences according to sex.

Our study suggest sex as a key factor for inequalities, which increased with time in relation to birth outcomes. In contrast, in mental pathologies the opposite effect was seen, with with girls presenting decreasing inequalities. We have not found any previous studies that have made estimates of this kind, and so we are unable to offer comparisons. We think that sex should be systematically included as a key interaction factor in studies of health inequalities.

The increase in inequalities with age may be partly explained by the cumulative impact of the socioeconomic situation affecting the child population during the 2014–2021 period. For respiratory conditions and injuries an age effect was found, with fewer inequalities in early childhood and more during adolescence; this is probably because in early childhood higher SEP parents have a lower threshold for noticing health symptoms and for taking their child to the doctor [52]. The increasing relationship between vulnerabilities (i.e., low household income, violence, material deprivation, and so on) and children's health as they grow older has frequently been described [52]. There is evidence [52, 53] that inequalities emerge during adolescence due to the cumulative SEP effect; in diseases with a chronic profile such as respiratory disorders, congenital anomalies and mental and behavioural disorders, the cumulative effect of comorbidity and the difficulty of cure means that the pathology tends to be concentrated in low SEP populations, and increases over time. Diseases and adverse events with an acute profile (i.e., injuries) may experience an increase in inequalities in adolescence due to a cumulative effect of adversities or poor parenting behaviours over the course of childhood [11, 38, 52, 53].

### Strenghts and limitations

Healthcare administrative data are highly sensitive to barriers to the health system, and the restrictions imposed at the time of the pandemic have had an impact on the number of contacts. The data should therefore be interpreted with caution, since in certain years they may not represent the true burden of disease.

This study included a broad representation of childhood pathology, comprising ten diseases and adverse events with different etiologies associated with the greatest inequalities in childhood. To our knowledge this is the first study to assess trends in inequalities in childhood in a set of diseases and adverse events in Catalonia, and to enable comparisons between diseases and sexes. This approach adds robustness, given that all the population is affected by the same common external factors.

The study used individual-level data from more than 1.8 million individuals, which allowed information on the economic and employment status of at least one of a child’s parents or guardians to be linked with the child’s health status. The assignment of the legal guardian (father or mother) is arbitrary, although it is often related to the one with the lower income, as this way pharmaceutical copayment will be lower for the children. So, the data do not provide complete information about household economic status, although in the very low SEP category does capture the unit family socioeconomic position as it includes people who live in a household with any member employed or receiving welfare support (i.e. in risk of social exclusion or poverty). Also a more detailed categorisation of SEP would also have been desirable. Even though this approach has been used successfully in many previous studies [6, 15–17].

The recording of the medical diagnoses mainly rely on interactions within the public healthcare system, although previous diseases not diagnosed in the public system are also recorded. Even in settings with universal healthcare access, there is a coexistence of private and public health services, particularly among high SEP households. It is estimated that 30% of children under 15 years old use both private and public healthcare services, with 88% belonging to medium and high SEP categories. On the other hand, 74% of all contacts in healthcare (85% in emergency units) are performed in the public system [54]. This dual usage may contribute to an underreporting of certain diseases, particularly those that are more common, less severe, such as respiratory diseases or mental health.

All source of information used (RCA and CMBD) are considered to be of high quality, for three reasons. First, they include the entire population of Catalonia given that all residents are granted universal healthcare coverage (99.6% of all residents are registered). Second, they have a validation system, and third, they contain historical patient data on diagnoses.

Furthermore, the RII is one of the most frequently used indices used in epidemiology for the measurement of socioeconomic inequalities in health. It takes account of all socioeconomic levels and their relative weight in the population, rather than comparing only the two most extreme SEP levels. Finally, we also estimated mean increases to help policy makers prioritise actions for the diseases with the most pronounced trends in inequality.

## Conclusion

Although trends in inequality in childhood do not increase in all diseases, our results suggest that health inequalities among children in Catalonia are not declining and are unlikely to do so in the coming years [28, 29]. The efforts at government level to tackle inequalities [55] have not produced any tangible results. Public health preventive programmes and interventions designed to modify social behaviour are less effective in low income groups and have a lower impact on health outcomes [56]. This means that more attention must be paid to the design of interventions and to determining who benefits from them.

Is the social welfare structure working [57]? At present, it is not reducing health inequalities in children; deprived children continue to present poorer health results. Until we focus on tackling social and economic disparities, health inequalities will persist or will even continue to grow.

### Supplementary Information

**Supplementary Material 1.**

**Supplementary Material 2.**

**Supplementary Material 3.**

**Supplementary Material 4.**

## Data Availability

CMBD is an administrative registry constructed and fed with the information that all centres send in to the Administration. The Administration automatically dissociates any personal information from the rest, and assigns a randomly generated number to each case. This register is used by the Administration to plan healthcare services, and to assess the quality of the care provided. The anonymised and unidentified data at individual level will be accessible to the research staff of the research centres accredited by the Research Centres of Catalonia (CERCA) institution https://cerca.cat/en/, Catalonia healthcare public agents (Catalan acronym SISCAT) https://catsalut.gencat.cat/ca/coneix-catsalut/presentacio/model-sanitari-catala/siscat/, and public university research centres based in Catalonia, as well as the same health administration.

## Abbreviations

EU-28: European Union-28
AROPE: Rate at risk of poverty and social exclusion
SEP: Socioeconomic position
Catalan acronym RCA: Central Registry of Insured Persons
Catalan acronym CMBD: Registry of the Minimum Basic Dataset
RII: Relative index of inequality
Ridit-score: Socioeconomic weighted rank
