# Supplementary material for: Health inequalities in childhood diseases: temporal trends in the inter-crisis period
Source: Int J Equity Health. 2024 Apr 17;23:76. doi: 10.1186/s12939-024-02169-5 (PMC11025183; doi:10.1186/s12939-024-02169-5)
Supplement: Supplementary file 1 — Supplementary Material 1. [file 12939_2024_2169_MOESM1_ESM.docx]

**Supplementary figure 1. Frequency trends by disease/adverse event according to children’s age. (Boys: straight lines; Girls: dashed lines)**

1. **Diseases/adverse events with higher frequencies.**

1. **Diseases/adverse events with lower frequencies**

Notes: Congenital (congenital anomalies), Adverse birth outcomes (short gestation, low birth weight, fetal growth retardation) and anxiety disorders (adaptive and anxiety disorders).
