# Supplementary material for: Health inequalities in childhood diseases: temporal trends in the inter-crisis period
Source: Int J Equity Health. 2024 Apr 17;23:76. doi: 10.1186/s12939-024-02169-5 (PMC11025183; doi:10.1186/s12939-024-02169-5)
Supplement: Supplementary file 2 — Supplementary Material 2. [file 12939_2024_2169_MOESM2_ESM.docx]

**Supplementary Table 1. Clinical pathology codes included in each group disease.**

| **Disease variable** | **CCS** | **CCSR** | **ICD-10-MC** | **ICD-9-MC** |
| --- | --- | --- | --- | --- |
| Asthma | 128 | RPS009 | J45(20-22), J45(30-32), J45(40-42), J45(50-52), J459(01-02), J45909, J459(90-91), J45998 |  |
| Bronquitis | 127 | RSP005, 008 | J20, J21 | 466 |
| Adjustment and anxiety disorders | 650, 651 | MBD007,005 | F064, F10(180,280), F10980, F12(180,280, 980), F13(180,280, 980), F14(180,280, 980), F15(180,280,980), F16(180,280,980), F18(180,280,980), F19(180,280,980), F40(00-02,10,11,210,218,220,228,230-233, 240-243,248,290,291,298, 8, 9), F41(0,1,3,8,9), F43(0,10-12,20-25,29, 80,81,89,90),F44(0-2,4-7,81,89,9), F481,F930,F940,F941,F942 |  |
| Mood disorders | 657 | MBD002-004,008 | F06(30-32,34), F32(0-4,8,80,81,89,9,A), F33(0-3,41,8,9), F34(1,81,89,9), F39, F530, F602, F63(1,2,81,89,9), F91(0-3,8,9), O906 |  |
| Injuries | 225-236, 239-240, 244 | INJ001-19,  INJ125-27,  INJ038-56,  INJ062-64,  INJ073 | S(00-99), T(07,15-28,30-34,78,79) |  |
| Poisoning | 241-243 | INJ022-24,  INJ059-61 | T(66-78,80-88) |  |
| Adverse birth outcomes: short gestation, low birth weight, foetal growth retardation | 219 | PNL002 | P(05-07) |  |
| Congenital anomalies | 213-217 | MAL001-010 | Q(00-09) |  |
| Obesity |  | >+2SD (more than two standard deviation above the median in WHO growth reference for children and adolescents)^α^ | | |
| Overweight |  | >+1SD (more than one standard deviation above the median in WHO growth reference for children and adolescents)^α^ | | |

^α^WHO. Growth reference data for 5-19 years old. Available: <https://www.who.int/tools/growth-reference-data-for-5to19-years>

Abreviations: CCS Clinical Classifications Software, CCSR Clinical Classifications Software Refined, ICD-9-MC International Classification of Diseases, 9th revision Clinical modification, ICD-10-MC International Classification of Diseases, 10th revision Clinical modification, SD Standard deviation
