# Supplementary material for: Health inequalities in childhood diseases: temporal trends in the inter-crisis period
Source: Int J Equity Health. 2024 Apr 17;23:76. doi: 10.1186/s12939-024-02169-5 (PMC11025183; doi:10.1186/s12939-024-02169-5)
Supplement: Supplementary file 3 — Supplementary Material 3. [file 12939_2024_2169_MOESM3_ESM.docx]

**Supplemetary Table 2. Relative Index of Inequality (RII), temporal trend by sex and interaction trend#sex, 2014–2021.**

|  |  | **RII (95% CI)** |  |  |  |  |  |  |  |  | **Temporal trend** | | **Interaction trend#sex** | |
| --- | --- | --- | --- | --- | --- | --- | --- | --- | --- | --- | --- | --- | --- | --- |
| **Disease*** | **Sex** | **2014** | **2015** | **2016** | **2017** | **2018** | **2019** | **2020** | **2021** |  | **β** | **p-value†** | **β** | **p-value†** |
| **Asthma** | Boys | 1.19  (1.09, 1.29) | 1.20  (1.17, 1.23) | 1.20  (1.17, 1.22) | 1.26  (1.22, 1.3) | 1.26  (1.21, 1.30) | 1.26  (1.25, 1.26) | 1.26  (1.24, 1.29) | 1.24  (1.22, 1.26) |  | 0.009 | 0.005 | -0.0002 | 0.973 |
|  | Girls | 1.30  (1.29, 1.31) | 1.31  (1.27, 1.34) | 1.38  (1.28, 1.48) | 1.45  (1.40, 1.50) | 1.40  (1.32, 1.48) | 1.39  (1.32, 1.45) | 1.41  (1.37, 1.46) | 1.36  (1.21, 1.51) |  | 0.009 | 0.023 |  |  |
| **Bronchitis** | Boys | 1.26  (1.22, 1.30) | 1.30  (1.28, 1.31) | 1.32  (1.28, 1.36) | 1.31  (1.31, 1.32) | 1.30  (1.28, 1.33) | 1.28  (1.26, 1.31) | 1.40  (1.34, 1.46) | 1.31  (1.22, 1.39) |  | 0.004 | 0.052 | 0.001 | 0.790 |
|  | Girls | 1.29  (1.24, 1.34) | 1.31  (1.28, 1.33) | 1.36  (1.32, 1.40) | 1.38  (1.36, 1.40) | 1.35  (1.34, 1.35) | 1.30  (1.24, 1.37) | 1.46  (1.40, 1.51) | 1.37  (1.30, 1.43) |  | 0.004 | 0.091 |  |  |
| **Injuries** | Boys | 1.30  (1.29, 1.32) | 1.33  (1.31, 1.36) | 1.33  (1.3, 1.35) | 1.37  (1.37, 1.38) | 1.39  (1.36, 1.42) | 1.38  (1.35, 1.41) | 1.39  (1.34, 1.45) | 1.42  (1.41, 1.43) |  | 0.019 | ≤0.001 | 0.004 | 0.080 |
|  | Girls | 1.28  (1.24, 1.31) | 1.32  (1.31, 1.33) | 1.33  (1.27, 1.38) | 1.32  (1.32, 1.32) | 1.38  (1.33, 1.44) | 1.38  (1.3, 1.45) | 1.41  (1.38, 1.44) | 1.42  (1.42, 1.42) |  | 0.023 | ≤0.001 |  |  |
| **Poisoning** | Boys | 1.22  (1.01, 1.44) | 1.07  (0.82, 1.33) | 1.28  (1.18, 1.38) | 1.52  (1.26, 1.78) | 1.80  (1.62, 1.98) | 2.05  (1.85, 2.25) | 2.06  (1.45, 2.66) | 1.77  (1.60, 1.93) |  | 0.100 | ≤0.001 | -0.024 | 0.251 |
|  | Girls | 1.33  (1.19, 1.46) | 1.41  (1.36, 1.46) | 1.69  (1.17, 2.20) | 1.44  (1.28, 1.60) | 1.90  (1.49, 2.31) | 2.33  (2.11, 2.56) | 2.04  (1.85, 2.23) | 2.29  (2.06, 2.52) |  | 0.082 | ≤0.001 |  |  |
| **Mood disorders** | Boys | 2.03  (1.52, 2.55) | 2.04  (1.89, 2.19) | 2.34  (2.11, 2.58) | 2.07  (2.04, 2.10) | 2.21  (1.87, 2.55) | 2.22  (2.12, 2.31) | 2.27  (2.03, 2.51) | 2.34  (2.05, 2.63) |  | 0.021 | 0.014 | -0.039 | ≤0.001 |
|  | Girls | 2.34  (2.04, 2.64) | 2.49  (2.42, 2.55) | 2.57  (2.47, 2.66) | 2.41  (2.29, 2.53) | 2.51  (2.20, 2.81) | 2.40  (1.97, 2.83) | 2.31  (2.19, 2.43) | 2.12  (2.05, 2.2) |  | -0.019 | 0.012 |  |  |
| **Adjustment and anxiety disorders** | Boys | 2.51  (2.17, 2.84) | 2.62  (2.51, 2.74) | 2.78  (2.51, 3.05) | 2.66  (2.63, 2.69) | 2.76  (2.70, 2.83) | 2.71  (2.58, 2.84) | 2.60  (2.30, 2.90) | 2.38  (2.14, 2.62) |  | 0.009 | 0.103 | -0.028 | ≤0.001 |
|  | Girls | 2.65  (2.37, 2.92) | 2.62  (2.31, 2.94) | 2.84  (2.76, 2.91) | 2.73  (2.48, 2.99) | 2.72  (2.71, 2.74) | 2.58  (2.17, 2.98) | 2.51  (2.49, 2.52) | 2.14  (2.05, 2.23) |  | -0.019 | ≤0.001 |  |  |
| **Congenital anomalies** | Boys | 1.34  (1.22, 1.46) | 1.47  (1.37, 1.56) | 1.21  (1.09, 1.33) | 1.32  (1.20, 1.43) | 1.37  (1.29, 1.45) | 1.52  (1.50, 1.55) | 1.97  (1.89, 2.05) | 1.27  (1.20, 1.34) |  | 0.012 | 0.033 | 0.024 | 0.003 |
|  | Girls | 1.06  (1.04, 1.09) | 1.15  (1.01, 1.29) | 0.89  (0.83, 0.96) | 1.15  (1.11, 1.19) | 1.26  (1.14, 1.38) | 1.34  (1.32, 1.35) | 1.77  (1.73, 1.80) | 1.15  (1.07, 1.23) |  | 0.033 | ≤0.001 |  |  |
| **Adverse birth outcomes** | Boys | 2.66  (1.86, 3.45) | 1.92  (1.74, 2.10) | 1.44  (0.89, 1.99) | 2.14  (2.00, 2.28) | 2.43  (2.34, 2.51) | 2.33  (2.00, 2.66) | 3.86  (2.86, 4.87) | 1.70  (1.41, 1.98) |  | 0.009 | 0.550 | 0.062 | 0.003 |
|  | Girls | 1.77  (1.25, 2.29) | 1.82  (1.76, 1.87) | 1.38  (1.06, 1.70) | 2.65  (1.97, 3.34) | 2.34  (2.20, 2.49) | 2.86  (1.41, 4.32) | 4.78  (4.21, 5.36) | 1.83  (1.46, 2.21) |  | 0.071 | ≤0.001 |  |  |
| **Overweight^¥^** | Boys | 1.17  (1.14, 1.20) | 1.23  (1.18, 1.28) | 1.32  (1.29, 1.36) | 1.32  (1.15, 1.49) | 1.40  (1.39, 1.42) | 1.43  (1.33, 1.53) | 1.37  (1.33, 1.42) | 1.47  (1.47, 1.47) |  | 0.037 | ≤0.001 | 0.004 | 0.499 |
|  | Girls | 1.38  (1.32, 1.44) | 1.43  (1.38, 1.48) | 1.45  (1.31, 1.60) | 1.51  (1.40, 1.61) | 1.57  (1.47, 1.67) | 1.72  (1.53, 1.90) | 1.60  (1.49, 1.70) | 1.77  (1.75, 1.79) |  | 0.041 | ≤0.001 |  |  |
| **Obesity** | Boys | 2.28  (2.19, 2.37) | 2.53  (2.36, 2.71) | 2.61  (2.52, 2.70) | 2.79  (2.73, 2.85) | 3.06  (2.94, 3.17) | 3.33  (3.04, 3.61) | 3.06  (2.92, 3.19) | 3.37  (3.19, 3.54) |  | 0.057 | ≤0.001 | -0.009 | 0.171 |
|  | Girls | 2.75  (2.51, 2.99) | 2.77  (2.62, 2.92) | 3.07  (2.99, 3.15) | 3.05  (3.02, 3.09) | 3.32  (3.1, 3.54) | 3.42  (3.42, 3.42) | 3.45  (3.23, 3.67) | 3.77  (3.76, 3.79) |  | 0.049 | ≤0.001 |  |  |

Note: Temporal trend: obtained by performed a generalised linear model (log-binomial regression) with a logarithmic link function with a two-way interaction term between the Ridit-score and year for each sex. Interaction trend#sex: obtained by performed a generalised linear model (log-binomial regression) with a logarithmic link function with a three-way interaction term between the Ridit-score, sex and year.

*Children population included: From 0 to 14 years old: asthma, bronchitis, injuries and poisoning. From 0 to 2 years old: congenital anomalies and adverse birth outcomes (short gestation, low birth weight and foeatl growth retardation). From 5 to 14 years old: mood disorders, adjustment and anxiety disorders, overweight and obesity.

^¥^Overweight does not include obesity.

^†^Significant at 95% Confidence Level.
