# Supplementary material for: Health inequalities in childhood diseases: temporal trends in the inter-crisis period
Source: Int J Equity Health. 2024 Apr 17;23:76. doi: 10.1186/s12939-024-02169-5 (PMC11025183; doi:10.1186/s12939-024-02169-5)
Supplement: Supplementary file 4 — Supplementary Material 4. [file 12939_2024_2169_MOESM4_ESM.docx]

**Supplementary Table 3. Relative Index of Inequality (RII), temporal trend by age and trend#age interaction, 2014–2021.**

|  |  | **RII (95% CI)** |  |  |  |  |  |  |  |  | **Temporal trend** | | **Interaction trend#age** | |
| --- | --- | --- | --- | --- | --- | --- | --- | --- | --- | --- | --- | --- | --- | --- |
| **Disease/adverse event*** | **Age** | **2014** | **2015** | **2016** | **2017** | **2018** | **2019** | **2020** | **2021** |  | **β** | **p-value†** | **β** | **p-value†** |
| **Asthma** | <2 | 1.12  (0.78, 1.46) | 1.03  (1.03, 1.03) | 1.00  (0.77, 1.22) | 1.02  (0.84, 1.21) | 0.89  (0.66, 1.11) | 0.87  (0.82, 0.92) | 1.05  (0.84, 1.27) | 0.89  (0.66, 1.11) |  | -0.036 | ≤0.001 | 0.022 | ≤0.001 |
|  | 2-5 | 1.14  (0.98, 1.30) | 1.22  (1.21, 1.24) | 1.32  (1.14, 1.50) | 1.35  (1.25, 1.46) | 1.23  (1.03, 1.42) | 1.22  (1.20, 1.24) | 1.01  (0.95, 1.08) | 1.16  (1.14, 1.18) |  | -0.010 | 0.131 |  |  |
|  | 6-10 | 1.29  (1.26, 1.33) | 1.29  (1.25, 1.34) | 1.29  (1.24, 1.34) | 1.40  (1.26, 1.53) | 1.40  (1.30, 1.50) | 1.38  (1.37, 1.39) | 1.46  (1.43, 1.49) | 1.45  (1.44, 1.46) |  | 0.019 | ≤0.001 |  |  |
|  | 11-14 | 1.19  (1.15, 1.22) | 1.20  (1.12, 1.28) | 1.25  (1.21, 1.30) | 1.30  (1.26, 1.34) | 1.34  (1.33, 1.35) | 1.35  (1.25, 1.44) | 1.36  (1.30, 1.41) | 1.34  (1.31, 1.37) |  | 0.023 | ≤0.001 |  |  |
| **Bronchitis** | <2 | 1.05  (1.00, 1.11) | 1.11  (1.09, 1.14) | 1.14  (1.13, 1.14) | 1.04  (1.00, 1.09) | 1.03  (1.02, 1.03) | 1.01  (1.01, 1.02) | 1.12  (1.10, 1.15) | 1.03  (1, 1.05) |  | -0.012 | ≤0.001 | 0.008 | ≤0.001 |
|  | 2-5 | 1.44  (1.41, 1.47) | 1.41  (1.41, 1.42) | 1.46  (1.37, 1.54) | 1.57  (1.53, 1.60) | 1.50  (1.38, 1.62) | 1.38  (1.35, 1.40) | 1.33  (1.30, 1.36) | 1.49  (1.36, 1.61) |  | 0.005 | 0.112 |  |  |
|  | 6-10 | 1.66  (1.63, 1.69) | 1.65  (1.63, 1.66) | 1.66  (1.63, 1.69) | 1.61  (1.56, 1.65) | 1.69  (1.65, 1.73) | 1.69  (1.51, 1.88) | 1.75  (1.68, 1.81) | 1.71  (1.68, 1.75) |  | 0.015 | ≤0.001 |  |  |
|  | 11-14 | 1.85  (1.77, 1.93) | 1.93  (1.72, 2.13) | 1.81  (1.79, 1.83) | 1.67  (1.59, 1.75) | 1.85  (1.85, 1.86) | 1.69  (1.34, 2.05) | 1.72  (1.68, 1.76) | 1.67  (1.49, 1.84) |  | -0.003 | 0.601 |  |  |
| **Injuries** | <2 | 1.15  (1.11, 1.19) | 1.15  (1.11, 1.18) | 1.07  (1.07, 1.08) | 1.18  (1.12, 1.24) | 1.14  (1.11, 1.17) | 1.13  (1.12, 1.15) | 1.11  (1.08, 1.14) | 1.24  (1.20, 1.29) |  | 0.005 | 0.133 | 0.005 | ≤0.001 |
|  | 2-5 | 1.21  (1.16, 1.27) | 1.22  (1.21, 1.23) | 1.22  (1.20, 1.23) | 1.25  (1.17, 1.33) | 1.31  (1.16, 1.45) | 1.24  (1.23, 1.24) | 1.27  (1.25, 1.28) | 1.24  (1.22, 1.26) |  | 0.010 | 0.001 |  |  |
|  | 6-10 | 1.24  (1.20, 1.27) | 1.29  (1.26, 1.33) | 1.31  (1.27, 1.35) | 1.27  (1.21, 1.33) | 1.32  (1.30, 1.34) | 1.36  (1.35, 1.36) | 1.34  (1.33, 1.35) | 1.42  (1.42, 1.42) |  | 0.020 | ≤0.001 |  |  |
|  | 11-14 | 1.55  (1.51, 1.58) | 1.59  (1.52, 1.66) | 1.56  (1.51, 1.61) | 1.61  (1.58, 1.64) | 1.65  (1.61, 1.69) | 1.60  (1.56, 1.64) | 1.69  (1.62, 1.76) | 1.71  (1.68, 1.75) |  | 0.023 | ≤0.001 |  |  |
| **Poisoning** | <2 | 1.35  (1.18, 1.52) | 1.63  (0.85, 2.41) | 1.44  (1.24, 1.65) | 1.34  (1.3, 1.38) | 1.81  (1.06, 2.57) | 2.56  (2.52, 2.59) | 1.38  (0.75, 2.00) | 1.85  (1.40, 2.30) |  | 0.042 | 0.039 | 0.014 | 0.184 |
|  | 2-5 | 1.46  (1.26, 1.66) | 1.23  (1.22, 1.25) | 1.47  (1.44, 1.51) | 1.75  (1.60, 1.90) | 1.97  (1.62, 2.31) | 1.90  (1.16, 2.64) | 1.71  (1.36, 2.05) | 2.39  (0.64, 4.14) |  | 0.073 | ≤0.001 |  |  |
|  | 6-10 | 0.96  (0.96, 0.96) | 0.90  (0.74, 1.05) | 1.24  (1.02, 1.46) | 1.20  (0.92, 1.48) | 1.70  (0.99, 2.40) | 1.80  (0.82, 2.78) | 2.08  (1.06, 3.10) | 1.24  (0.96, 1.52) |  | 0.095 | ≤0.001 |  |  |
|  | 11-14 | 1.64  (1.10, 2.17) | 1.46  (1.17, 1.75) | 1.83  (1.57, 2.08) | 1.80  (1.47, 2.12) | 1.90  (1.07, 2.73) | 2.51  (2.14, 2.88) | 2.98  (1.89, 4.07) | 2.55  (2.52, 2.59) |  | 0.086 | ≤0.001 |  |  |
| **Mood disorders** | 6-10 | 1.87  (1.78, 1.96) | 1.81  (1.80, 1.81) | 2.33  (2.04, 2.61) | 1.80  (1.7, 1.91) | 2.13  (2.07, 2.18) | 2.00  (1.6, 2.4) | 1.95  (1.67, 2.23) | 2.23  (2.04, 2.42) |  | 0.015 | 0.084 | -0.028 | 0.013 |
|  | 11-14 | 2.43  (2.21, 2.66) | 2.65  (2.63, 2.66) | 2.50  (2.49, 2.51) | 2.56  (2.36, 2.77) | 2.50  (2.46, 2.53) | 2.51  (2.29, 2.74) | 2.49  (2.36, 2.61) | 2.19  (2.13, 2.25) |  | -0.013 | 0.070 |  |  |
| **Adjustment and anxiety disorders** | 6-10 | 2.39  (2.19, 2.59) | 2.30  (2.27, 2.34) | 2.68  (2.50, 2.86) | 2.41  (2.10, 2.71) | 2.57  (2.41, 2.73) | 2.50  (2.40, 2.61) | 2.29  (2.03, 2.55) | 2.26  (2.02, 2.49) |  | 0.002 | 0.748 | -0.013 | 0.107 |
|  | 11-14 | 2.71  (2.53, 2.88) | 2.89  (2.87, 2.92) | 2.86  (2.75, 2.97) | 2.89  (2.87, 2.92) | 2.84  (2.70, 2.99) | 2.72  (2.62, 2.83) | 2.71  (2.49, 2.93) | 2.22  (2.11, 2.32) |  | -0.011 | 0.037 |  |  |
| **Congenital anomalies** | <2 | 1.21  (1.19, 1.24) | 1.36  (1.32, 1.40) | 1.23  (1.22, 1.23) | 1.27  (1.27, 1.27) | 1.43  (1.40, 1.46) | 1.62  (1.60, 1.63) | 1.90  (1.88, 1.93) | 1.32  (1.27, 1.36) |  | 0.030 | ≤0.001 | ------ | ------- |
| **Adverse birth outcomes** | <2 | 2.29  (2.02, 2.55) | 2.05  (1.69, 2.41) | 1.91  (1.88, 1.94) | 2.61  (2.58, 2.64) | 2.73  (2.49, 2.96) | 3.17  (2.73, 3.61) | 4.43  (3.38, 5.48) | 2.01  (1.93, 2.10) |  | 0.047 | ≤0.001 | ------ | ------- |
| **Overweight^¥^** | 6-10 | 1.19  (1.15, 1.24) | 1.22  (1.17, 1.28) | 1.28  (1.25, 1.31) | 1.33  (1.29, 1.37) | 1.38  (1.33, 1.42) | 1.49  (1.42, 1.56) | 1.35  (1.27, 1.44) | 1.50  (1.50, 1.51) |  | 0.035 | ≤0.001 | 0.005 | 0.353 |
|  | 11-14 | 1.46  (1.41, 1.50) | 1.55  (1.52, 1.59) | 1.63  (1.46, 1.80) | 1.59  (1.55, 1.64) | 1.71  (1.6, 1.81) | 1.72  (1.68, 1.76) | 1.84  (1.73, 1.95) | 1.89  (1.83, 1.95) |  | 0.041 | ≤0.001 |  |  |
| **Obesity** | 6-10 | 2.33  (2.21, 2.45) | 2.41  (2.27, 2.55) | 2.63  (2.49, 2.77) | 2.72  (2.69, 2.74) | 3.02  (2.84, 3.20) | 3.27  (3.14, 3.40) | 3.09  (3.06, 3.11) | 3.41  (3.37, 3.45) |  | 0.057 | ≤0.001 | -0.018 | 0.010 |
|  | 11-14 | 2.89  (2.58, 3.19) | 3.37  (3.32, 3.42) | 3.24  (3.10, 3.38) | 3.39  (3.02, 3.75) | 3.49  (3.41, 3.57) | 3.56  (3.54, 3.59) | 3.59  (3.52, 3.66) | 3.92  (3.82, 4.01) |  | 0.039 | ≤0.001 |  |  |

Note: Temporal trend: obtained by performing a generalised linear model (log-binomial regression) with a logarithmic link function and a two-way interaction term between the Ridit-score and year for each age category. trend#age Interaction: obtained by performing a generalised linear model (log-binomial regression) with a logarithmic link function and a three-way interaction term between the Ridit-score, age and year.

*Diseases/adverse events included per age group: From 0 to 14 years old: asthma, bronchitis, injuries and poisoning. From 0 to 2 years old: congenital anomalies and adverse birth outcomes (short gestation, low birth weight and fetal growth retardation). From 5 to 14 years old: mood disorders, adjustment and anxiety disorders, overweight and obesity.

^¥^Overweight does not include obesity.

^†^Significant at 95% confidence level.
